# Lemierre’s Syndrome: Case Presentation of a Rare and Possibly Life-Threatening Condition

**DOI:** 10.3390/medicina57101102

**Published:** 2021-10-14

**Authors:** Lucian Giubelan, Livia Dragonu, Vlad Pădureanu, Alexandru Neacșu, Mirela Mănescu, Andreea Cristina Stoian, Florentina Dumitrescu

**Affiliations:** 1 Department of Infectious Disease, Faculty of Medicine, University of Medicine and Pharmacy of Craiova, 200349 Craiova, Romania; ligiubelan@yahoo.com (L.G.); livia_dragonu@yahoo.com (L.D.); andreea_plr@yahoo.com (A.C.S.); dumitrescu_florentina@yahoo.com (F.D.); 2 “Victor Babeș” Hospital of Infectious Diseases and Pulmonology from Craiova, 200515 Craiova, Romania; manescumirela@yahoo.com; 3 Department of Internal Medicine, Faculty of Medicine, University of Medicine and Pharmacy of Craiova, 200349 Craiova, Romania

**Keywords:** Lemierre’s syndrome, oropharyngeal infection, jugular thrombophlebitis, venous thrombosis, infectious diseases

## Abstract

Lemierre’s syndrome is, presently, a very rare condition, but a life-threatening one. The syndrome was first described in 1936 by Andre Lemierre and comprises an oropharyngeal infection (most commonly associated with anaerobic bacteria *Fusobacterium necrophorum*), internal jugular vein thrombophlebitis and, possibly, secondary septic metastasis (common sites are lungs or brain). We describe such a rare case diagnosed at our Infectious Diseases Department in September 2019.

## 1. Introduction

In 1936, Andre Lemierre, a French physician, described 20 cases of anaerobic septicemia (18 deaths) [1]. The syndrome (named after him) comprises an initial site of infection in the oropharyngeal region and, subsequently, internal jugular vein thrombophlebitis; through the bloodstream, germs may be transported to other locations and generate septic metastases (mainly to the brain and lungs, but also to the kidneys or joints). Most cases involve young healthy patients. However, variants have been described that are atypical in the age of onset or in the location of the infection or septic metastases [2]. *Fusobacterium necrophorum* is the most isolated germ responsible for this syndrome [3]; however, other bacteria may be encountered.

## 2. Case Report

PCM, 18 years old, female, from the Gorj County, was admitted on 21 September 2019 to the Infectious Diseases Department with high fever (39–40 °C), chills, intense sore throat and headache. The onset of the disease was two weeks earlier with fever and sore throat. She had consulted the general practitioner who established the diagnosis of exudative tonsillitis and recommended treatment with 625 mg amoxicillin/clavulanate three times per day and 200 mg ibuprofen two times per day. The patient followed the treatment for 12 days, but the condition worsened, the fever gradually went up and a bulge appeared on the left side of the neck.

Her past medical history was inconspicuous; the patient denied consumption of tobacco, alcohol, recreational drugs or oral birth control drugs. She had received all the mandatory immunization (according to the Romanian national program).

There was no contact with other sick persons with similar symptoms.

When she was admitted to the hospital, her general condition was consistent with moderate sepsis; she had fever (38.9 °C), elevated pulse rate (137 bpm), normal blood pressure (116/74 mm Hg) and normal oxygen saturation (97%). A significant lymph nodes enlargement (about 6 cm in diameter) was noted on the left side of the neck, in front and behind the sternocleidomastoid muscle, and left supraclavicular adenopathy; there was limited movement of the enlarged lymph nodes and they were hard on palpation. Both tonsils showed white exudate on their surface. There was no abnormal lung or heart sounds, no liver or spleen enlargement and no neurological abnormalities. The urine output was normal.

The initial laboratory tests are shown in Table 1.

**Figure 1:**
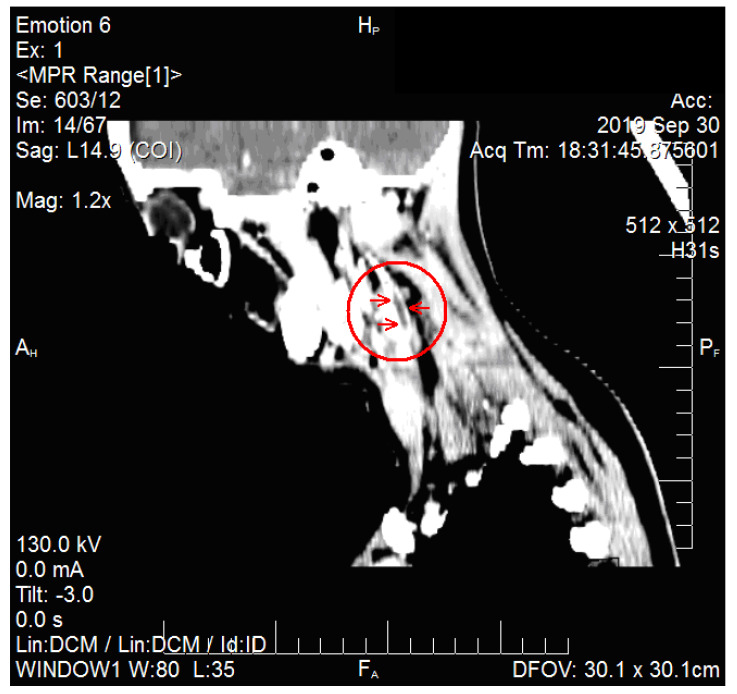
Patient PCM, CT of the neck, sagittal view, sign of internal jugular vein thrombosis (circle and arrows).

**Figure 2:**
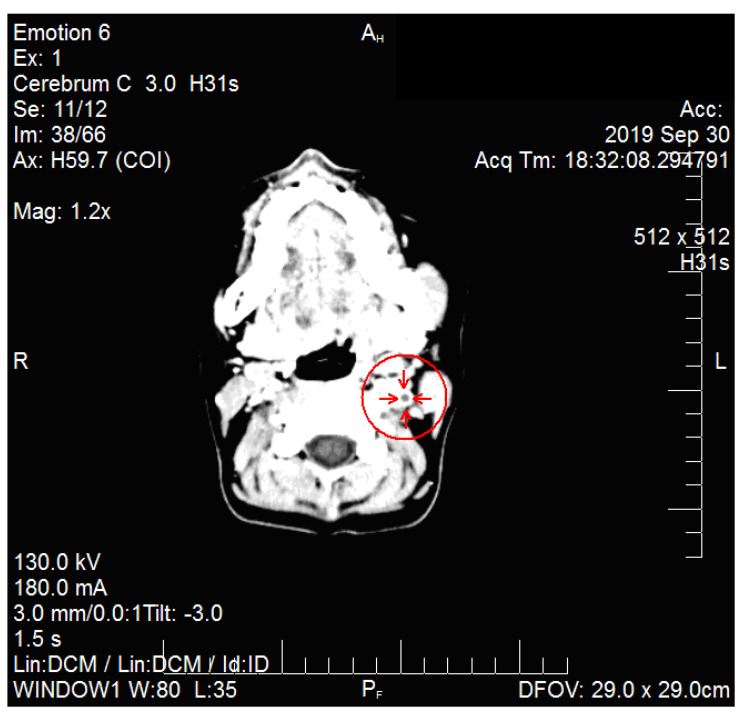
Patient PCM, CT of the neck, axial view, sign of internal jugular vein thrombosis (circle and arrows).

The chest X-ray showed consolidation on the lower right lobe. An ultrasound of the left side of the neck highlighted the lymph nodes enlargement, intense vascularization and a deep edema. A computed tomography (CT) of the neck (see Figure 1 and Figure 2) and the upper thorax showed segmental partial thrombosis of the left internal jugular vein. The Ear-Nose-Throat (ENT) examination established the diagnosis of cryptic tonsillitis and cervical adenitis.

The throat swab culture on blood agar media and blood culture using BACTEC vials for aerobic germs were negative. An enzyme-linked immunosorbent assay (ELISA) test for HIV was also negative.

The diagnosis of Lemierre’s syndrome was established and the initial prognosis was reserved (the patient met the old criteria for sepsis); she started antimicrobial treatment with penicillin G, 4 international megaunits (MUI)/day, intravenous (i.v.), and metronidazole, 1.5 g/day, per os (p.o.). She also received enoxaparin, 0.6 mL twice a day, s.c., and diclofenac, 100 mg/day, p.o.

The fever gradually decreased, the patient returned to normal temperature after 6 days of treatment, and the general condition improved; however, the lymph nodes remained enlarged throughout the period of hospital admission.

The follow-up laboratory tests are shown in Table 2.

The patient was discharged after 16 days; she received a prescription for metronidazole, 1.5 g/day, p.o., for another 14 days and for rivaroxaban, 20 mg/day, p.o., for 30 days; she continued with this regimen at home as recommended.

A month later, she was seen in our department: she was in good condition, there were no signs of infection, but the ultrasound still showed signs of thrombosis of the jugular vein. She refused the surgical intervention to remove the thrombus.

## 3. Discussion

Today, Lemierre’s syndrome is a rare condition (1 case per million annually) [2,4], probably due to the extensive use of antimicrobials. This was the first case diagnosed in our clinic since its opening in 1984 and probably one of the first descriptions of this condition in a Romanian patient. A search on PubMed and Medscape returned only one result of a fellow Romanian diagnosed and treated in Greece [4].

From the clinical point of view, our case is a classic one, but we were not able to detect the causative germ, probably because only aerobic culture media were used and prior antimicrobial therapy was administered. Unfortunately, anaerobic culture media were not available at the hospital at that time. However, given the fact that the starting point was exudative pharyngitis, we suspected bacterial involvement, which is considered sufficient according to the contemporary diagnostic criteria [5]. 

A number of alternative diagnoses were taken into account prior to the laboratory examinations. First, we considered streptococcal tonsillitis complicated with a left peritonsillar abscess, but there was no trismus or tonsillar protrusion. Infectious mononucleosis might lead to exudative pharyngitis, but the lymph nodes should be bilaterally enlarged and much smaller; also, a rash should be noted after the treatment with aminopenicillin. Given the aspect of the lymph nodes, there was another idea of leukemia or malignant lymphoma. There are many cases of tuberculosis in Romania, but oral involvement, usually due to *Mycobacterium bovis*, is extremely rare, and the girl denied consumption of raw milk. Diphtheria has not been observed at our clinic for the last 40 years and the patient was immunized against it. We also dismissed the possibility of oropharyngeal anthrax or tonsillar cancer due to human papillomavirus (HPV).

After the diagnosis of Lemierre’s syndrome, the initial prognosis was reserved due to the mortality rate which even today remains significant (4–25% [4,5,6,7,8,9]). A recent meta-analysis found a mortality rate of 4% [10]. Our case also met the 2001 criteria of sepsis [11,12,13].

The patient was initially treated with amoxicillin/clavulanate and there are three probable causes why there was no good result: either non-susceptible bacteria were involved or the dose was insufficient or the antimicrobial was not able to properly penetrate the tonsillar crypts. Based on the available literature data, we empirically chose the antimicrobial combination of penicillin and metronidazole, with good clinical and biological results. The duration of the treatment varies from 2 to 6 weeks according to the medical literature [2,4,5,6,7]; in our case, it was about 6 weeks (including pre- and post-hospitalization).

Another problem was whether or not to use anticoagulation. We found arguments for and against it in some case reports or small case series [2,3,4,5,7,8,9,10,14]. A recent meta-analysis from 2020 that collected the data of 712 patients diagnosed with Lemierre’s syndrome found that in 5.2% of the cases, the patients experienced new venous thromboembolism, in 11.7% of the cases—new peripheral septic lesions; however, the rates of both events were lower if anticoagulation was used; the number of bleeding events was found to be low [10,12,15]. Our choice was to use enoxaparin concomitant with antimicrobials (which should kill any bacteria potentially released due to the lysis of the thrombus). Because the thrombus persisted and the girl refused surgical intervention, she continued therapy with rivaroxaban, which is more convenient to use at home, having an oral route of administration.

Our report includes information on the course of blood tests during the clinical course of Lemierre’s syndrome that have not been described by most previous reports. Similarly to other forms of non-septic venous thrombophlebitis, D-dimers were considerably elevated upon diagnosis; while this may have been due to sepsis, this finding suggests that the clinical value of this parameter in supporting diagnosis should be explored by future research. In addition, we analyzed laboratory parameters over the course of hospitalization, showing the timing of their progressive improvement in the case in which both antimicrobial and anticoagulant treatments were initiated. They can be used as reference for comparison with cases in which only antimicrobial treatment was used.

## 4. Conclusions

Lemierre’s syndrome is a very rare disease with reserved prognosis and a significant mortality rate which requires personalized therapy with an antimicrobial combination in association with anticoagulants.

## Figures and Tables

**Table 1:** Patient PCM, the initial laboratory tests (September 21).

Laboratory Test	Value	Reference Range
Hemoglobin (g/L)	11.3	11–15
White blood cells count (×10^3^/mm^3^)	21.4	4–9
Neutrophils (%)	81.2	50–68
Thrombocytes (×10^3^/mm^3^)	498	150–300
Erythrocyte sedimentation rate (mm/1 h)	120	12–20
Urea (mg/dL)	23.5	20–40
Glucose (mg/dL)	91.7	80–115
TGP (u/L)	14.9	10–35
Urinalysis	normal	–
Fibrinogen (mg/dL)	550	200–400
C-reactive protein (mg/L)	192	<10
Procalcitonin (ng/mL, semiquantitative)	20	<1
Quick time (s)	11.4	11–18
International normalized ratio	1.01	1–2
D-dimers (ng/mL)	2300	<250

**Table 2:** Laboratory investigations during follow-up visits.

Laboratory Test	Day 11 of Admission	Day 16 of Admission
White blood cells count (×10^3^/mm^3^)	9.4	7.8
Neutrophils (%)	58	61
Bands (%)	–	–
Thrombocytes (×10^3^/mm^3^)	521	417
Erythrocyte sedimentation rate (mm/1 h)	66	59
Fibrinogen (mg/dL)	266	–
C-reactive protein (mg/L)	12	–
Procalcitonin (ng/mL, semiquantitative)	<1	–
